# In-Bag Morcellation as a Routine for Laparoscopic Hysterectomy

**DOI:** 10.1155/2017/6701916

**Published:** 2017-11-29

**Authors:** Stefan Rimbach, Miriam Schempershofe

**Affiliations:** ^1^Department of Gynecology and Obstetrics, Agatharied Hospital, Norbert-Kerkel-Platz, 83734 Hausham, Germany; ^2^Academic Teaching Hospital, Ludwig-Maximilian University of Munich (LMU), Munich, Germany

## Abstract

Tissue morcellation during laparoscopic hysterectomy carries the risk of spreading cells from unsuspected malignancy. Contained morcellation inside a bag is supposed to minimize this risk. The present study evaluated routine use of in-bag morcellation during laparoscopic hysterectomy in a consecutive patient cohort (*n* = 49). The system used was More-Cell-Safe (A.M.I. Austria). Median age was 47 (35 to 76) years and BMI 25.1 (18.8 to 39.8). Indications for hysterectomy were fibroids (71.4%), adenomyosis (16.3%), prolapse (8.2%), and bleeding disorders (4.1%). 48 (98%) patients underwent supracervical hysterectomy and 1 (2%) underwent total hysterectomy. No unsuspected malignancy occurred. Median weight of extirpated tissue was 195 g (18 to 1110). Residual tissue and/or fluid in the bag amounted to 29 g (0 to 291). Median overall duration of surgeries was 100.5 min, and median time associated with the use of the bag was 10 min (5 to 28), significantly correlated with uterine volume (*p* = 0.0094) and specimen weight (*p* = 0.0002), but not with patient's BMI (*p* = 0.6970). Technical success rate for contained morcellation was 93.9%. Peritoneal washings after contained morcellation were all negative for malignant or smooth muscle cells.

## 1. Introduction

Laparoscopic hysterectomy requires special techniques in order to remove the uterine specimen from the peritoneal cavity despite small incisions. In case of supracervical hysterectomy, the main alternative to salvage laparotomy consists in tissue morcellation, which may also become necessary for total hysterectomy specimens when they are too large for being pulled through the vagina entirely. Since 1993, method of choice for tissue morcellation has been power morcellation [1], which came under scrutiny after strong and repeated warnings by the FDA in 2014 [2, 3], indicating the risk for spreading malignant cells during the procedure originating from unsuspected sarcoma mistaken for benign fibroids.

There is an ongoing debate on incidence and significance of the phenomenon, with wide ranges of reported frequencies between 0.02–0.25% referring to unsuspected leiomyosarcoma and 0.13–0.47% to uterine malignancies overall [4–6]. But beside malignant processes, so-called parasitic leiomyoma or peritoneal adenomyosis has been reported as a result of tissue dissemination as well [7–9].

Regardless of the dispute on incidence numbers, international gynecological societies strongly recommend thorough patient information on the potential risks and consent before considering morcellation [4, 5, 10]. Beyond counseling, risk stratification might improve patient selection, but despite upcoming recommendations [4] valid concepts are missing so far.

Another strategy to optimize patient safety, simultaneously maintaining the proven advantages of minimal invasive hysterectomy [11, 12], would consist in improving surgical techniques of morcellation. A promising approach could be contained morcellation inside a bag in order to prevent spilling of uterine tissue or cells [13, 14]. In this regard, we recently introduced a new system for in-bag morcellation, which proved feasibility and a preventive effect in both experimental and clinical pilot settings [15, 16].

The aim of the present work was to report our continued experience using the new system in clinical routine application during laparoscopic hysterectomy.

## 2. Materials and Methods

### 2.1. Patients

Experiences with contained morcellation during laparoscopic hysterectomy in *n* = 49 consecutive patients were retrospectively analysed in this observational cohort study after obtaining CE approval. Informed consent was obtained from all patients. Counseling included information on the surgical techniques, alternatives, risks, and benefits of laparoscopic hysterectomy as well as on power morcellation risks. Possible spread of undetected malignancy was quoted according to the statement of the DGGG [5].

### 2.2. Operative Procedure

**Figure 1:**
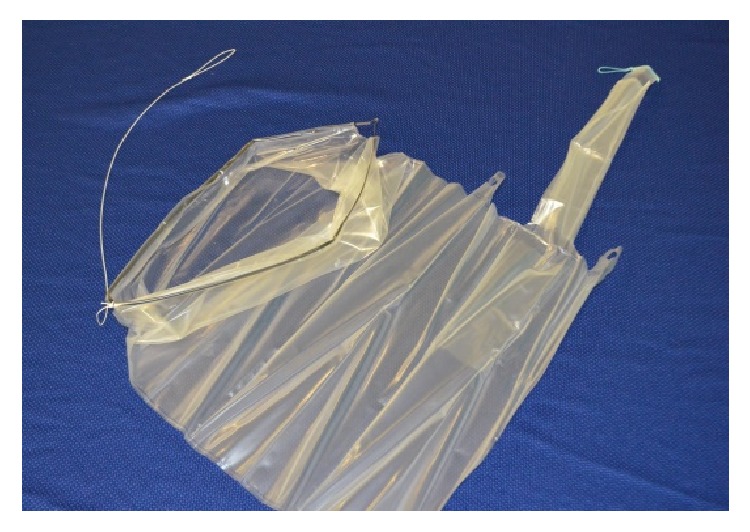
More-Cell-Safe bag (A.M.I. Austria) for contained power morcellation: material polyurethane, feed sizes of 340 × 250 mm, capacity 2.5 liters; large opening of 160 mm for specimen placement and morcellator access and small tubular opening for optic trocar access.

According to the description in our preceding pilot study, the procedure started with total or supracervical laparoscopic hysterectomy. A multiport approach was used with a 11 mm umbilical trocar for optic (0°), suprapubic, and two lateral 6 mm trocars. After completing the hysterectomy, the More-Cell-Safe system (A.M.I. Austria) for contained power morcellation (Figure 1) was introduced into the peritoneal cavity via either a 12 mm disposable plastic sheath (MCS-Port, A.M.I. Austria) or a 13 mm reusable metal trocar (Karl Storz, Germany). The polyurethane bag (More-Cell Bag, A.M.I. Austria) has feed sizes of 340 × 250 mm and a capacity of 2.5 liters. It has two openings measuring 160 and 16 mm. The large opening serves as a specimen placement and morcellator access suprapubically. The small opening allows optical trocar insertion at the umbilical site. The outer part of the tubular bag opening is everted to protect it from contamination by spread cells during use. In order to allow the bag to be introduced through the suprapubic trocar, it is delivered folded into a sleeve, that has to be stripped while inserting into the bag. First-generation bags were rolled instead of being folded. These were applied in 11 cases. Second-generation bags are folded allowing self-opening of the large mouth of the bag into the peritoneal cavity after insertion. Those were applied in all consecutive cases (12–49).

An 11 mm sleeve (Visi-Shield) covers the optic in order to prevent cell contamination during in-bag use. The shield is disposed at the end of morcellation before further use of the optic inside the peritoneal cavity.

After inserting the bag into the peritoneal cavity, the specimen was placed into the bag with the help of grasping forceps using the lateral trocars. For larger specimen handling, a third instrument was applied using the suprapubic access port. After positioning the specimen, the large mouth of the bag was pulled out suprapubically with help of a retrieval thread and simultaneously the respective port removed. The tubular bag opening was now exteriorized through the umbilical access while removing the 11 mm optic trocar.

**Figure 2:**
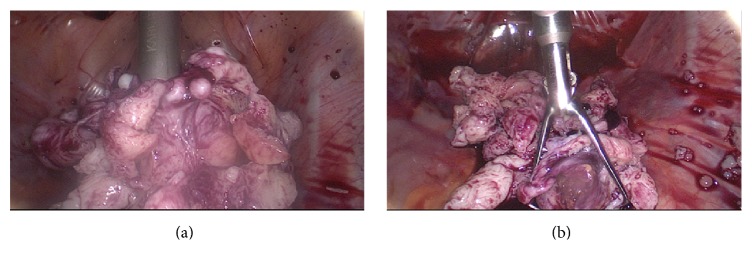
(a/b) Technique of contained in-bag power morcellation of a supracervical hysterectomy specimen using More-Cell-Safe (A.M.I., Austria).

At this point, the umbilical fascia was enlarged in order to allow entering the larger trocar into the bag. Trocar size is determined by the Visi-Shield diameter requiring at least 12 mm trocar to allow its passage and adequate gas flow between shield and trocar wall. Fascia enlargement was performed bluntly digitally in the first 9 cases and with sharp cutting with scissors under external vision in the 40 consecutive cases. For optic trocar access to the bag, a 12 mm blunt disposable trocar (Versaport, Covidien Medtronic) was used in the first 17 patients, and in all consecutive patients a 13 mm reusable blunt tipped metal trocar was used (Karl Storz, Germany). A pseudopneumoperitoneum was now established inflating the bag via the umbilical trocar and the optic reentered covered by its protective shield. The morcellator (Rotocut G1, Karl Storz Germany, 12 or 15 mm according to the surgeons preference) was then entered bluntly into the bag at its suprapubic opening and electromorcellation started in a contained, but otherwise unchanged technique (Figure 2). In case of total hysterectomy, vaginal closure was performed prior to morcellation.

After completion, both morcellator and optic were withdrawn from the bag. Visi-Shield and optic trocar were disposed, the everted tubular part of the bag was unrolled, and the bag was securely closed by two knots. Now, the bag was removed by manually pulling it towards the suprapubic site. For continued laparoscopy, the previously removed 11 mm optic trocar and the optic without shield was reinserted.

### 2.3. Parameter and Statistical Analysis

Patient baseline characteristics were recorded including age, BMI, history of previous surgery, and preoperative findings including transvaginal ultrasound measurements and indication for surgery.

Procedural parameters were recorded as type of hysterectomy, eventual complementary surgery, and duration of surgery, differentiated into overall procedure time, times associated with the use of the bag, and morcellation time. Overall resected tissue weight was calculated from measured weights of morcellated specimen and residual tissue or fluid inside the bag after removal.

Complications were recorded as intra- or postoperative ones, and duration of hospital stay was registered from surgery to discharge.

Feasibility of in-bag morcellation was determined as intraoperative technical success rate defined as successfully completed contained morcellation procedure including proof of bag integrity after removal by visual inspection and blue stain fluid filling. Additional qualitative parameter was bag handling, adequate pseudopneumoperitoneum, in-bag visualization, and morcellation performance as assessed by the surgeon.

Postoperative histology was registered and compared to preoperative findings. In order to evaluate potential cell spillage from morcellated tissue, cytology was analysed for the presence of smooth muscle cells from peritoneal washings taken at the end of the surgical procedure.

Statistical analyses were performed using GraphPad software with descriptive calculations of median and minimum to maximum range, mean, and 95% confidence interval. Linear regression analysis was performed to describe influence factors on bag associated duration of surgery.

## 3. Results

### 3.1. Patient Baseline Characteristics (Tables 1 and 2)

Median age was 47, ranging from 35 to 76 (mean 48.3; 95% CI 45.93–50.64). Patient BMI ranged from 18.8 to 39.8 with a median at 25.1 (mean 26.7; 95% CI 25.04–28.41).

Only 24 (49%) patients had no previous abdominal surgery. The others had undergone one or more previous surgeries, among these laparoscopic interventions in 18 (36.7%) cases (myomectomy, endometriosis, ovarian cystectomy or ovariectomy, ectopic pregnancy, tubal ligation, cholecystectomy, and sigma resection), cesarean sections in 10 (20.4%) cases, and open surgery in 8 (16.3%) cases (endometriosis, appendectomy, cholecystectomy, and adhesiolysis for bowel obstruction).

Indications for hysterectomy were symptomatic fibroids in 35 (71.4%) cases (7 isolated, 28 multiple myoma), adenomyosis in 8 (16.3%) cases, prolapse in 4 (8.2%) cases (combined with cervicosacropexy), and bleeding disorders in the presence of dehiscent cesarean uterotomy in 2 (4.1%) cases. Three (8.6%) of the 35 myoma patients were pretreated with ulipristal acetate.

Median uterine volume based on transvaginal ultrasound measurement was 350 cm^3^ (range 36 to 2016; mean 515.2; 95% CI 390.05–640.28).

### 3.2. Procedural Parameter (Table 3)

Scheduled laparoscopic hysterectomy was performed in all patients without intraoperative complications. 48 (98%) underwent supracervical hysterectomy and 1 (2%) underwent total hysterectomy.

All patients underwent additional interventions (46 salpingectomy, 1 ovarian cyst removal, 4 endometriosis resection, and 4 cervicosacropexy) besides hysterectomy, which were however performed before in-bag morcellation in all cases except one umbilical hernia repair. Remarkably, in 2 of the three cases after previous laparoscopic myomectomy, there were several parasitic myoma cases. All were removed with unsuspicious histological results.

**Figure 3:**
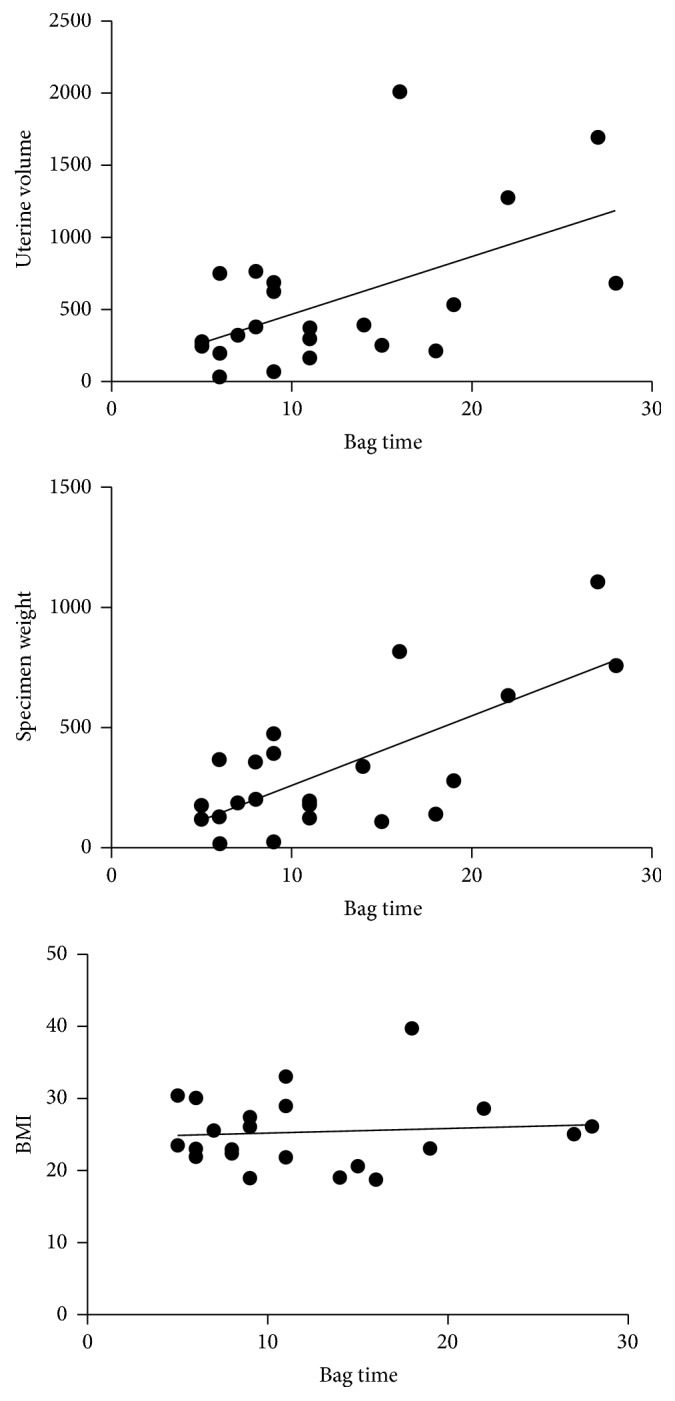
Linear regression analysis showing bag associated time during surgery significantly correlated with uterine volume (*p* = 0.0094) and specimen weight (*p* = 0.0002), but not with patients BMI (*p* = 0.6970).

Median overall duration of surgeries was 100.5 min (range 55 to 239; mean 110.4; 95% CI 97.45–123.38). The median overall time frame of bag use from insertion to removal was 19.5 min (range 8 to 82; mean 24.4; 95% CI 16.85–31.97). Morcellation time ranged from 2 to 54 min with a median of 9 min (mean 12.1; 95% CI 7.15–17.12). The time associated with the use of the bag (in total, before and after morcellation) was 10 min (median; range 5 to 28; mean 12.3; 95% CI 9.23–15.31). There was a significant correlation with uterine volume (*p* = 0.0094) and specimen weight (*p* = 0.0002), but not with patient's BMI (*p* = 0.6970) (Figure 3).

Inserting and preparation of the bag including specimen placement until start of morcellation took 8.5 min (median; range 4 to 26; mean 11.0; 95% CI 8.33–13.67). Removal of the bag after morcellation was performed in 1 min (median; range 0 to 8; mean 1.3; 95% CI 0.53–2.01).

Median overall weight of extirpated tissue was 195 g ranging from 18 to 1110 (mean 269.7; 95% CI 204.20–335.27). From this, morcellated tissues had a median weight of 170 g (range 18 to 819; mean 250; 95% CI 191.41–308.59). Residual tissue and/or blood that remained in the bag and then removed at the end of the procedure amounted to 29 g (median; range 0 to 291; mean 47.8; 95% CI 15.60–80.10).

### 3.3. Clinical Complications and Duration of Hospital Stay

There were no intraoperative clinical complications. Postoperatively, one patient developed a fever of unknown origin but was treated successfully with antibiotics. Median duration until discharge from the hospital was 3 days (range 2 to 11; mean 3.3; 95% CI 2.91–3.66).

### 3.4. Feasibility of In-Bag Morcellation (Table 4)

Contained morcellation was performed successfully and intact bag after removal in 46 of 49 cases resulted in a technical success rate of 93.9%.

In 3 (6.1%) cases bags appeared not intact after the procedure. One bag showed a 3 mm tear at its tubular part during postprocedural extracorporeal stain fluid filling test. Pattern and localisation of the lesion as well as documented technical problems during umbilical trocar insertion lead to the assumption of shearing by the trocar passing through a too small fascia incision. A second bag was first punctually damaged with the morcellator forceps before morcellation, removed, and replaced by another bag, but this finally ruptured during forced extraction with an inside remaining calcified 5 cm myoma not passing through an inadequate suprapubic incision. Comparably, the third bag, which resulted in defect, ruptured during forced extraction because of an ignored residual piece of myoma of 30 × 15 × 15 mm.

Despite successful procedures, handling difficulties occurred in 8 (16.3%) cases. In two cases, the surgeon decided to remove and replace the initial bags because they were twisted after insertion to the peritoneal cavity and specimen placement was not possible. In both cases, first-generation bags were applied; procedures were successful during second attempt. In a third case, the bag was perforated with the umbilical trocar during insertion. The perforation occurred before morcellation and was immediately detected when the optic was introduced, showing the peritoneal cavity outside the bag. The bag was removed and replaced. The second attempt was then completely successful after having enlarged the umbilical fascia opening. A fourth case also required change of the bag because of an apparent defect at the tubular segment after pulling it through the umbilical trocar before even starting the in-bag procedure. In the fifth and sixth case with handling difficulties, several attempts of optic trocar insertion into the bag were necessary and only successful after adequately enlarging the fascia incision. In two further difficult cases, it was the surgeon who forgot to strip the sleeve from the bag during initial insertion, which made complete removal and reinsertion of the bags necessary.

There were no difficulties regarding pseudopneumoperitoneum and during actual morcellation. Once the optic trocar was adequately positioned, a pseudopneumoperitoneum could be established regularly in all cases. This applied as well for the defect bag presumably damaged by the optic trocar prior to morcellation. In-bag visualization was not impaired and allowed regular power morcellation in all cases. Median morcellation time was 9 min (range 2 to 54; mean 12.1; 95% CI 7.15 to 17.12).

### 3.5. Postoperative Histology and Results of Peritoneal Washings

Fibroids were found in 35 cases, sometimes associated with adenomyosis. Adenomyosis alone was described in 11 cases. In three cases, specimen did not show pathology except dehiscence of a former cesarean uterotomy scar in 2 instances. Histology confirmed the clinical suspicion in 45 (91.8%) cases. In 1 case an adenomyoma had been mistaken for a fibroid, and in 3 (6.1%) cases histology revealed a fibroid (1) and adenomyosis (2) sonographically were not detected. Cases of unsuspected malignancies did not occur.

Cytology from the peritoneal washings showed mesothelial cells and/or leucocytes, erythrocytes, and macrophages in all examined cases but was negative for malignant or smooth muscle cells each time.

## 4. Discussion

Technical feasibility of in-bag power morcellation using the setting here applied had previously been shown in a small pilot series [16]. Data from the present collection of 49 consecutive patients were gathered in order to prospectively examine suitability for use in clinical routine. In fact, enclosed patients represent an unselected cohort of women at varying age, BMI, medical history, and indications for hysterectomy.

Technical success was achieved under these conditions in 93.9%, confirming our pilot results. The absence of smooth muscle cells in all peritoneal washings suggests effective prevention against spilling of cells from the morcellated tissue [15, 17].

The occurrence of three failure cases (6.1%) indicates a remaining risk potential that needs being addressed during informed consent. Analysis of the cases reveals the surgeon's impact on outcome. Restricting the incision of the fascia too much at the umbilical trocar site was not only the main reason for handling difficulties during bag preparation, but also responsible for one of the failure cases with a trocar associated shear lesion of the bag. Bag ruptures occurred only as a consequence of forced extraction in the presence of larger in-bag residuals. Meticulous removal of morcellated tissue from the bag and avoiding force during bag extraction will make bag rupture less likely. In case of tissue remnants which are resistant to morcellation, such as a calcified myoma in one of our cases, adequate abdominal wall incision is mandatory.

Small residual tissue pieces and fluid in the bag, however, do not interfere with appropriate extraction. In our series, they were regularly noted with a median weight of 29 g (range 0 to 291 g). Anapolski et al. [18] reported 12.1 g (range 7 to 19 g) from a pilot study using a different type of bag. Saving time and effort to collect these fragments and rinse the peritoneal cavity may be a positive side-effect of in-bag versus uncontained morcellation.

Nevertheless, application of a bag system consumes time, which was reported to prolong surgery by 20–30 min as compared to retrospective controls with uncontained morcellation [14, 19, 20]. In the present study, median total time associated with the bag use was only 10 min, confirming the positive experience from our pilot work [16]. It ranged from 5 to 28 min, which was significantly correlated with uterine size, ranging from 18 to 1110 g (median 195 g). The most time consuming stage was inserting the bag, placing the specimen, and preparing for morcellation with a median proportional time span of 8.5 min versus only 1 min to remove the bag afterwards. Time specifications in different studies must be compared with caution because of their correlation with specimen sizes in the respective cohorts. Nevertheless, two recent studies using different systems [18, 21] came to slightly longer, but essentially comparable results with 10.5 and 12.5 min preparation time for in-bag morcellation, thus increasing acceptability for clinical routine.

In contrast to our pilot experiences [16], uterine size was not limiting applicability of contained morcellation in this series, though the largest specimen weighed 1110 g. Despite the surgeon's subjective impression, there was no statistically detectable correlation of difficulties, measurable in procedure duration, with patient's BMI. Problems in handling occurred with first-generation bags, which did not open properly. This problem was solved by introducing differently folded second-generation bags, providing easier access for specimen placement. The importance of adequate umbilical fascia incision has already been pointed out. Once, blunt trocar access to the bag interior was successfully established, the actual morcellation process was assessed unhindered and not prolonged under the circumstances of the contained setting.

Technical alternatives consist in vaginal, single-site, or manual open concepts for in-bag morcellation [22–26]. Vaginal access would have been possible for only one of our patients undergoing total hysterectomy, while it was not applicable for all others, operated upon supracervically. Single-site techniques would require a major change of our clinical routine but have to be taken into consideration, as recently the FDA allowed marketing of a bag for contained morcellation using single-site access [27]. Manual in-bag morcellation requires appropriate incisions of the abdominal wall and abandons power morcellation in favor of the scalpel but represents a practicable alternative with the advantage of direct external visual control.

## 5. Conclusions

The technique presented here allows in-bag power morcellation during laparoscopic hysterectomy in a usual multiport approach with proven feasibility. Preventive effectiveness against spilling from morcellated tissue is suggested by reproducible results of negative peritoneal washings. The promising single center data of this study will now need confirmation in a prospective multicentric approach.

## Figures and Tables

**Table 1:** Indications for hysterectomy.

Symptomatic fibroids	35 (71.4%)
Adenomyosis	8 (16.3%)
Prolapse (combined with cervicosacropexy)	4 (8.2%)
Bleeding disorders (dehiscent cesarean scar)	2 (4.1%)

**Table 2:** Patient and specimen characteristics.

	Median	Range
Patient age	47 years	35–76
BMI	25.1	18.8–39.8
Uterine volume (ultrasound)	350 cm^3^	36–2016
Weight of extirpated tissue	195 g	18–1110
Weight of morcellated tissue	170 g	18–819
Residual tissue/fluid in the bag	29 g	0–291

**Table 3:** Duration of surgery, morcellation, and bag application.

	Median	Range
Overall duration of surgery	100.5 min	55–239
Overall time of bag use (in/out)	19.5 min	8–82
Morcellation time	9 min	2–54
Total time associated with bag use	10 min	5–28
Bag preparation time before morcellation	8.5 min	4–26
Bag removal time	1 min	0–8

**Table 4:** Technical feasibility of in-bag morcellation.

Successful and bag intact	46 (93.9%)

Bag defect	3 (6.1%)

Findings in cases of defect bag	(i) 3 mm tear at tubular part due to shearing by the umbilical trocar (too small fascia incision) (ii) Bag ruptured during forced extraction (calcified myoma 50 mm remaining in the bag) (iii) Bag ruptured during forced extraction (residual piece of myoma 30 mm ignored)

## References

[1] Steiner R. A., Wight E., Tadir Y., Haller U. (1993). Electrical cutting device for laparoscopic removal of tissue from the abdominal cavity. *Obstetrics and Gynecology*.

[2] http://www.fda.gov/MedicalDevices/Safety/AlertsandNotices/ucm393576.htm; last access 31.01.2017

[3] http://www.fda.gov/MedicalDevices/Safety/AlertsandNotices/ucm424443.htm; last access 31.01.2017

[4] Brölmann H., Tanos V., Grimbizis G. (2015). European society of gynaecological endoscopy (ESGE) steering committee on fibroid morcellation: options on fibroid morcellation: a literature review. *Journal of Gynecologic Surgery*.

[5] Beckmann M. W., Juhasz-Böss I., Denschlag D. (2015). Surgical methods for the treatment of uterine fibroids - risk of uterine sarcoma and problems of morcellation: position paper of the DGGG. *Geburtshilfe und Frauenheilkunde*.

[6] Bojahr B., De Wilde R. L., Tchartchian G. (2015). Malignancy rate of 10,731 uteri morcellated during laparoscopic supracervical hysterectomy (LASH). *Archives of Gynecology and Obstetrics*.

[7] Tan-Kim J., Hartzell K. A., Reinsch C. S. (2015). Uterine sarcomas and parasitic myomas after laparoscopic hysterectomy with power morcellation. *American Journal of Obstetrics & Gynecology*.

[8] Donnez O., Squifflet J., Leconte I., Jadoul P., Donnez J. (2007). Posthysterectomy pelvic adenomyotic masses observed in 8 cases out of a series of 1405 laparoscopic subtotal hysterectomies. *Journal of Minimally Invasive Gynecology*.

[9] Pereira N., Buchanan T. R., Wishall K. M. (2015). Electric morcellation-related reoperations after laparoscopic myomectomy and nonmyomectomy procedures. *Journal of Minimally Invasive Gynecology*.

[10] http://www.acog.org/Resources-And-Publications/Task-Force-and-Work-Group-Reports/Power-Morcellation-and-Occult-Malignancy-in-Gynecologic-Surgery

[11] Nieboer T. E., Johnson N., Lethaby A. (2009). Surgical approach to hysterectomy for benign gynaecological disease. *Cochrane Database Syst Rev*.

[12] Brown J. (2014). AAGL advancing minimally invasive gynecology worldwide: statement to the FDA on power morcellation. *Journal of Minimally Invasive Gynecology*.

[13] Cohen S. L., Einarsson J. I., Wang K. C. (2014). Contained power morcellation within an insufflated isolation bag. *Obstetrics & Gynecology*.

[14] Srouji S. S., Kaser D. J., Gargiulo A. R. (2015). Techniques for contained morcellation in gynecologic surgery. *Fertility and Sterility*.

[15] Rimbach S., Holzknecht A., Nemes C., Offner F., Craina M. (2015). A new in-bag system to reduce the risk of tissue morcellation: development and experimental evaluation during laparoscopic hysterectomy. *Archives of Gynecology and Obstetrics*.

[16] Rimbach S., Holzknecht A., Schmedler C., Nemes C., Offner F. (2016). First clinical experiences using a new in-bag morcellation system during laparoscopic hysterectomy. *Archives of Gynecology and Obstetrics*.

[17] Ikhena D. E., Paintal A., Milad M. P. (2016). Feasibility of washings at the time of laparoscopic power morcellation: a pilot study. *Journal of Minimally Invasive Gynecology*.

[18] Anapolski M., Panayotopoulos D., Alkatout I. (2016). Power morcellation inside a secure endobag: a pilot study. *Minimally Invasive Therapy & Allied Technologies*.

[19] Vargas M. V., Cohen S. L., Fuchs-Weizman N. (2015). Open power morcellation versus contained power morcellation within an insufflated isolation bag: comparison of perioperative outcomes. *Journal of Minimally Invasive Gynecology*.

[20] Winner B., Porter A., Velloze S., Biest S. (2015). Uncontained compared with contained power morcellation in total laparoscopic hysterectomy. *Obstetrics & Gynecology*.

[21] Paul P. G., Thomas M., Das T., Patil S., Garg R. (2016). Contained morcellation for laparoscopic myomectomy within a specially designed bag. *Journal of Minimally Invasive Gynecology*.

[22] Günthert A. R., Christmann C., Kostov P., Mueller M. D. (2015). Safe vaginal uterine morcellation following total laparoscopic hysterectomy. *American Journal of Obstetrics & Gynecology*.

[23] Aoki Y., Matsuura M., Matsuno T., Yamamoto T. (2016). Single-site in-bag morcellation achieved via direct puncture of the pneumoperitoneum cap, a cordless electric morcellator, and a 5-mm flexible scope. *European Journal of Obstetrics & Gynecology and Reproductive Biology*.

[24] Moawad G. N., Samuel D., Abi Khalil E. D. (2016). Abdominal Approaches to Tissue Containment and Extraction in Minimally Invasive Gynecologic Surgery. *Journal of Minimally Invasive Gynecology*.

[25] Serur E., Zambrano N., Brown K., Clemetson E., Lakhi N. (2016). Extracorporeal Manual Morcellation of Very Large Uteri Within an Enclosed Endoscopic Bag: Our 5-Year Experience. *Journal of Minimally Invasive Gynecology*.

[26] Venturella R., Rocca M. L., Lico D. (2016). In-bag manual versus uncontained power morcellation for laparoscopic myomectomy: randomized controlled trial. *Fertility and Sterility*.

[27] http://www.fda.gov/NewsEvents/Newsroom/PressAnnouncements/ucm494650.htm

